# Enhancing β-Carotene Concentration in Parental Lines of CO6 Maize Hybrid Through Marker-Assisted Backcross Breeding (MABB)

**DOI:** 10.3389/fnut.2020.00134

**Published:** 2020-10-14

**Authors:** Senthil Natesan, Thirusenduraselvi Duraisamy, Bharathi Pukalenthy, Sarankumar Chandran, Jagadeeshselvam Nallathambi, Karthikeyan Adhimoolam, Dhasarathan Manickam, Vellaikumar Sampathrajan, Samuel Jeberson Muniyandi, Laishram Joykumar Meitei, Nepolean Thirunavukkarasu, Ganesan Kalipatty Nalliappan, Ravikesavan Rajasekaran

**Affiliations:** ^1^Department of Plant Molecular Biology and Bioinformatics, Centre for Plant Molecular Biology and Biotechnology, Tamil Nadu Agricultural University, Coimbatore, India; ^2^Department of Seed Technology, Tamil Nadu Agricultural University, Coimbatore, India; ^3^Department of Plant Breeding and Genetics, Agricultural College and Research Institute, Tamil Nadu Agricultural University, Madurai, India; ^4^Department of Plant Biotechnology, Centre for Plant Molecular Biology and Biotechnology, Tamil Nadu Agricultural University, Coimbatore, India; ^5^Department of Biotechnology, Agricultural College and Research Institute, Tamil Nadu Agricultural University, Madurai, India; ^6^AICRP-MULLARP, Directorate of Research, Central Agricultural University, Imphal, India; ^7^Department of Genetics and Plant Breeding, College of Agriculture, Central Agricultural University, Imphal, India; ^8^Department of Genomics and Molecular Breeding, ICAR-Indian Institute of Millets Research, Hyderabad, India; ^9^Department of Plant Breeding and Genetics, Centre for Plant Breeding and Genetics, Tamil Nadu Agricultural University, Coimbatore, India; ^10^Department of Millets, Center for Plant Breeding and Genetics, Tamil Nadu Agricultural University, Coimbatore, India

**Keywords:** β-Carotene, Co6 maize hybrid, crtRB1, vitamin A deficiency, MABB

## Abstract

Vitamin A deficiency (VAD) is a global health problem; many people around the world, especially children and pregnant women, are VAD deficient or insufficient. Maize is known as an important source of provitamin A for humans. Hence, enhancement of provitamin A carotenoids (pVAC) in maize varieties through breeding or biofortification is a good option for alleviating VAD in developing countries, especially India. So far, numerous maize hybrids have been developed in India. Among them, CO6, derived from UMI1200 × UMI1230, is a popular maize hybrid and adapted to different agro-climatic zones of India, especially Tamil Nadu, a southern state of India. However, CO6 is deficient for pVAC carotenoid β-carotene. Thus, the objectives of this study were to increase the β-carotene concentration in UMI1200 and UMI1230 and generate the β-carotene enriched hybrids through marker-assisted backcross breeding (MABB). For this purpose, the maize genotype HP467-15 was used as the donor for transferring the β-carotene gene, *crtRB1*, into UMI1200 and UMI1230. In the MABB scheme, we used one gene-specific marker (*crtRB1* 3′TE) and 214 simples sequence repeat (SSR) markers for foreground and background selection, respectively. As a result, six improved lines with recurrent parent genome recovery (RPGR) ranging from 90.24 to 92.42%, along with good agronomic performance, were generated. The β-carotene concentration of the improved lines ranged from 7.056 to 9.232 μg/g. Furthermore, five hybrid combinations were generated using improved lines and evaluated in a comparative yield trial (CYT) and multi-location trials (MLT) along with the original hybrid CO6 and commercial hybrids. It was revealed that ACM-M13-002 was a superior hybrid with a 7.3-fold increase in β-carotene concentration and with a comparable yield to CO6. In summary, the improved maize inbreds can be used as possible donors for the development of β-carotene-rich cultivars in maize breeding programs and the β-carotene enriched hybrid developed in this study will hold great promise for food and nutritional security.

## Introduction

Vitamin A deficiency (VAD) is one of the most prevalent micronutrient deficiencies and poses a serious health problem to children and pregnant women around the world, especially in developing countries. The consequences of VAD include night blindness, reduced growth in children, and increased morbidity and mortality (1). Maize (*Zea mays* L.) is one of the major sources of provitamin A for humans. Thus, the improvement of provitamin A carotenoids (pVAC) in maize varieties through breeding or biofortification is a successful and cost-effective approach for alleviating VAD in developing countries, especially India (2). The preference of maize for biofortification also relies on higher bioavailability and bioconversion of pVAC into vitamin A. α-carotene, β-carotene, β-cryptoxanthin, zeaxanthin, and lutein are important carotenoid compounds that exist in maize grains (3). Among them, β-carotene has the highest provitamin A activity and can be converted easily to vitamin A by the human metabolism (4, 5). However, the success of this approach depends on the identification of superior maize genotypes with a high β-carotene concentration by characterizing the maize genotypes and subsequently transferring the high β-carotene from the superior genotypes to the popular maize cultivars through introgression breeding. However, β-carotene is present at a comparatively low level in maize kernels of popular cultivated lines, and only a very small percentage of maize varieties have naturally high β-carotene levels. Muthusamy et al. screened 105 maize inbreds of both Indian and CIMMYT origins for kernel β-carotene content. The results showed that kernel β-carotene concentration varied from 0.02 to 16.50 μg/g in maize inbreds (6). Hence, there is a need to understand the major genes involved in the carotenoid biosynthesis pathway that could be modulated for enhancing β-carotene concentration.

Recent advancements in maize genomics have reduced the breeding cycles and significantly increased the precision and effectiveness of breeding methods, and are also helping to identify the genes and biosynthesis pathways involved in β-carotene accumulation. Maize breeding programs through marker-assisted selection (MAS) have become more standard with the availability of reasonably dense molecular marker linkage maps, and many researchers have been pursuing the association of markers with known genes. Lycopene epsilon cyclase (*lcyE*) located at chromosome 8 and β-carotene hydroxylase enzyme (*crtRB1*) located at chromosome 10 are the two major genes associated with β-carotene accumulation in maize (3, 7). Among them, the gene *crtRB1* was stated to have a much greater effect on the concentrations of β-carotene than that of *LcyE* (8). The favorable allele, namely *crtRB1* 3'TE (allele 1, 543 bp), is accountable for causing a 2- to 10-fold upsurge in β-carotene concentration (8). Polymerase chain reaction (PCR) based co-dominant markers from the 3'TE region of the *crtRB1* gene provided opportunities to identify and develop the higher β-carotene concentration genotypes through MAS. Several studies showed the feasibility of this allele-based marker to detect the *crtRB1* allele in diverse maize genotypes (9–11).

In India, maize is one of the major cereal crops after rice and wheat, and is utilized as a major source of both food and feed. To date, several maize inbreds and hybrids have been developed for their economically important traits and are commercially available in India. Among them, CO6, derived from UMI1200 × UMI1230, is a popular single-cross hybrid and adapted to different agro-climatic zones of India, especially Tamil Nadu, a southern state of India. CO6 is well-suited for cultivation in both under irrigate and rainfed conditions and also exhibits multiple disease resistances. It shows superior performance in grain yield and quality, and ease in hybrid seed production (12). These salient features make CO6 a popular maize hybrid among farmers. However, CO6 is deficient in nutritional traits, especially β-carotene. Thus, it is necessary to improve the CO6 hybrid with a higher β-carotene concentration. Marker-assisted backcross breeding (MABB) is an effective approach to improve the nutritional traits in maize. Recently, several studies showed success with improving the nutritional traits in maize hybrids (Pusa Vivek QPM9 Improved, Pusa HM4 Improved, Pusa HM8 Improved, and Pusa HM9 Improved) using MABB (9, 13). With this background information, the present study aimed to: (i) introgress the *crtRB1* allele into the parental lines (UMI1200 and UMI1230) of CO6 hybrid using MABB; (ii) evaluate the improved lines agronomic performance and β-carotene concentration; and (iii) generate the hybrids using the improved lines and evaluate the hybrids in diverse maize growing regions of Tamil Nadu, a southern state of India.

## Materials and Methods

### Plant Genetic Materials

CO6 is a popular maize hybrid well-adapted to different agro-climatic zones of India, especially Tamil Nadu, a southern state of India. Maize inbreds viz., UMI1200 and UMI1230 are the parental lines of the CO6 hybrid. UMI1200 is a female parent for the CO6 hybrid and is suitable for tropical regions; it is characterized as a dent grain type. UMI1230 is a pollen parent for the CO6 hybrid and is suitable for both tropical and semi-arid regions. It is characterized as a flint type. Both have diverse genetic backgrounds and are placed in different subspecies in accordance with the starch type in the endosperm. Due to their excellent combining ability, these two inbreds were used to develop the CO6 hybrid. The donor parent was HP467-15 from CIMMYT (International Maize and Wheat Improvement Center, Mexico), which carries the *crtRB1* allele responsible for the high β-carotene concentration.

### Outline of the Conversion of Maize Inbreds to Its High β-Carotene Version

The conversion process included crossing, two generations of backcrossing, and two generations of selfing. All the field experiments were carried out on the Central farm, Tamil Nadu Agricultural University, Coimbatore, Tamil Nadu, India from 2011 to 2015. Foreground selection was done using a *crtRB1* gene-specific marker (*crtRB1* 3′TE) (7) to select the plants at F_1_, backcross, and selfed generations. Foreground selection targeted heterozygous plants with allele 1, 543 bp (Favorable allele) and allele 3, 296 bp (Unfavorable allele), or homozygous plants with allele 1, 543 bp (Favorable allele). Background selection was done to select the foreground positive plants with maximum recurrent parent genome recovery (RPGR) using polymorphic simple sequence repeat (SSR) markers in backcross and selfed generations. Two independent crosses, namely UMI1200 × HP467-15 and UMI1230 × HP467-15, were made during Rabi 2011-12 to produce F_1_ seeds. In Kharif 2012, the F_1_ plants were confirmed for their heterozygosity and backcrossed with the recurrent parent to produce BC_1_F_1_ progenies. In Rabi, 2012-13, the BC_1_F_1_ plants that were found to be heterozygous were backcrossed with the recurrent parent to produce BC_2_F_1_ progenies. Further, BC_2_F_1_ plants were screened to identify heterozygous and the selected plants were selfed to produce BC_2_F_2_ progenies during Kharif 2013. In Rabi 2013-14, BC_2_F_2_ progenies were screened before flowering to identify plants with allele 1, 543 bp (Favorable allele) and the selected plants were selfed to produce BC_2_F_3_ lines, whereas the homozygous plants with allele 3, 296 bp (Unfavorable allele) and heterozygous plants with a‘llele 1, 543 bp (Favorable allele) and allele 3, 296 bp (Unfavorable allele) were excluded. Agro-morphological character evaluation and β-carotene estimation were carried out in BC_2_F_3_ improved lines.

### Genomic DNA Isolation and Marker Genotyping

The genomic DNA was isolated from the young leaves using the cetyl trimethyl ammonium bromide (CTAB) method (14). The DNA was checked for its quantity and quality on 0.8% agarose gel. The *crtRB1* gene-specific marker (*crtRB1* 3′TE) (65F: ACACCACATGGACAAGTTCG, 62R: ACACTCTGGCCCATGAACAC, 66R: ACAGCAATACAGGGGACCAG) was used for foreground selection. A total of 214 simple sequence repeat (SSR) markers spanning uniformly across the maize genome were used for background selection. The primer sequences for SSR markers were obtained from the maize genome database (www.maizegdb.org) and were synthesized by Eurofins Ltd, Bangalore, India. The polymerase chain reaction (PCR) for *crtRB1* 3'TE/SSR markers and gel electrophoresis were carried out following the procedure of Muthusamy et al. and Pukalenthy et al. (9, 15). All the obtained genotyping results were tested for goodness of fit using the chi-square analysis. The amplicons of the markers were scored as “A” for the recurrent parent, “B” for the donor parent, and “H” for heterozygotes. Chi-square analysis was done using PROC MIXED of SAS and LSMEANS and ADJUST options were used to find the least square mean values and for the comparison of the *p-*value. The percentage of RPGR was calculated following the procedure of Sundaram et al. (16).

### Evaluation of Agro-Morphological Characters in Improved Lines

The recurrent and donor parents and the improved lines were evaluated for eight agro-morphological characters [plant height (cm), days to 50% tasselling (days), days to 50% silking (days), cob length (cm), number of kernel rows/ cob, number of kernels/row, grain yield/ plant (g), and 100-kernel weight (g)] which were recorded according to the plant stage based on the standard maize descriptors formulated by IBPGR (17).

### Estimation of β-Carotene Concentration Using HPLC Analysis

The seeds obtained from the recurrent and donor parents and the improved lines were subjected to β-carotene estimation. Since the carotenoid compounds are sensitive to light, the seeds were harvested at 14% moisture content and stored at 22–26°C. Estimation of β-carotene was carried out by adopting the Harvest plus protocol (18). The carotenoid compounds were extracted by grinding the seed samples using ice-cold acetone until a fine powder was obtained. Since the carotenoid compounds undergo photo-oxidation, further processing was done under yellow light (19, 20). The samples extracted were concentrated in a rotary evaporator at 45°C and made up to 2 ml using methanol prior to separation. The β-carotene concentration was quantified by high-performance liquid chromatography (HPLC). Samples were eluted by C18G 120A column (250 × 4.6 mm) and measured with a photodiode array detector set at 450 nm. The mobile phase comprised of Acetonitrile: Methanol: Ethyl acetate (80:10:10) at high pressure by the column with the flow rate of 1 mL min^−1^. β-carotene (M/s. Sigma Aldrich, India) reconstituted in methanol to five different concentrations (0.1 μg/g; 1 μg/g; 10 μg/g; 50 μg/g; 100 μg/g) was used to make the standard curve. The β-carotene concentration was identified by their characteristic spectra and comparison of their retention times with known standard solutions.

### Generation of Hybrids Using Improved Lines and Their Evaluation

Four improved lines from UMI1200 × HP467-15 and two improved lines from UMI1230 × HP467-15 were crossed during Kharif 2014 to generate the five hybrids. These hybrids were evaluated along with the commercial hybrid NK6240 and CO6 during the Rabi 2014-15 and Kharif 2015 at the Experimental Farm, Agricultural Research Station, Vaigai dam, Tamil Nadu Agricultural University, India and Maize Research Station, Tamil Nadu Agricultural University, Vagarai, India under comparative yield trial (CYT). Further, one superior hybrid identified from CYT was tested in multi-location trials (MLT) during Kharif 2016 and 2017 in diverse maize growing environments of Tamil Nadu along with eight maize hybrids including commercial hybrids. Kharif 2016 included four environments viz., Coimbatore (E1), Vagarai (E2), Vridhachalam (E3), and Bhavanisagar (E4) and Kharif 2017 included six environments viz., Coimbatore (E5), Vagarai (E6), Vridhachalam (E7), Bhavanisagar (E8), Vaigai Dam (E9), and Athiyandal (E10). Hybrids [namely NK6240 (G1), CO 6 (G2), ACM-M13-002 (G3), ACM-M13-007 (G4), CMH 11-583 (G5), VaMH 14020 (G6), VaMH 12014 (G7), 900 M GOLD (G8), and CMH 11-586 (G9)] were used in MLT. All the field trials were conducted in the irrigated condition. The spacing followed was 60 × 25 cm with a plot size of 5 × 3.6 m (6 rows each) and a fertilizer dosage of 250:75:75 kg/ha. Trials were carried out using a randomized complete block design (RCBD) with two replications. Grain yield data were subjected to a combined analysis of variance (ANOVA) using GEA-R statistical software (21). Additive Main Effect and Multiplicative Interaction (AMMI) model (22) and Genotype and Genotype by Environmental Interaction (GGE) effects biplot (23) were employed to analyse the Genotype by Environment (GE) interaction and to assess grain yield stability based on the principal component analysis (PCA).

## Results

### Marker-Assisted Introgression of the *crtRB1* Gene Into UMI1200 and UMI1230

**Figure 1:**
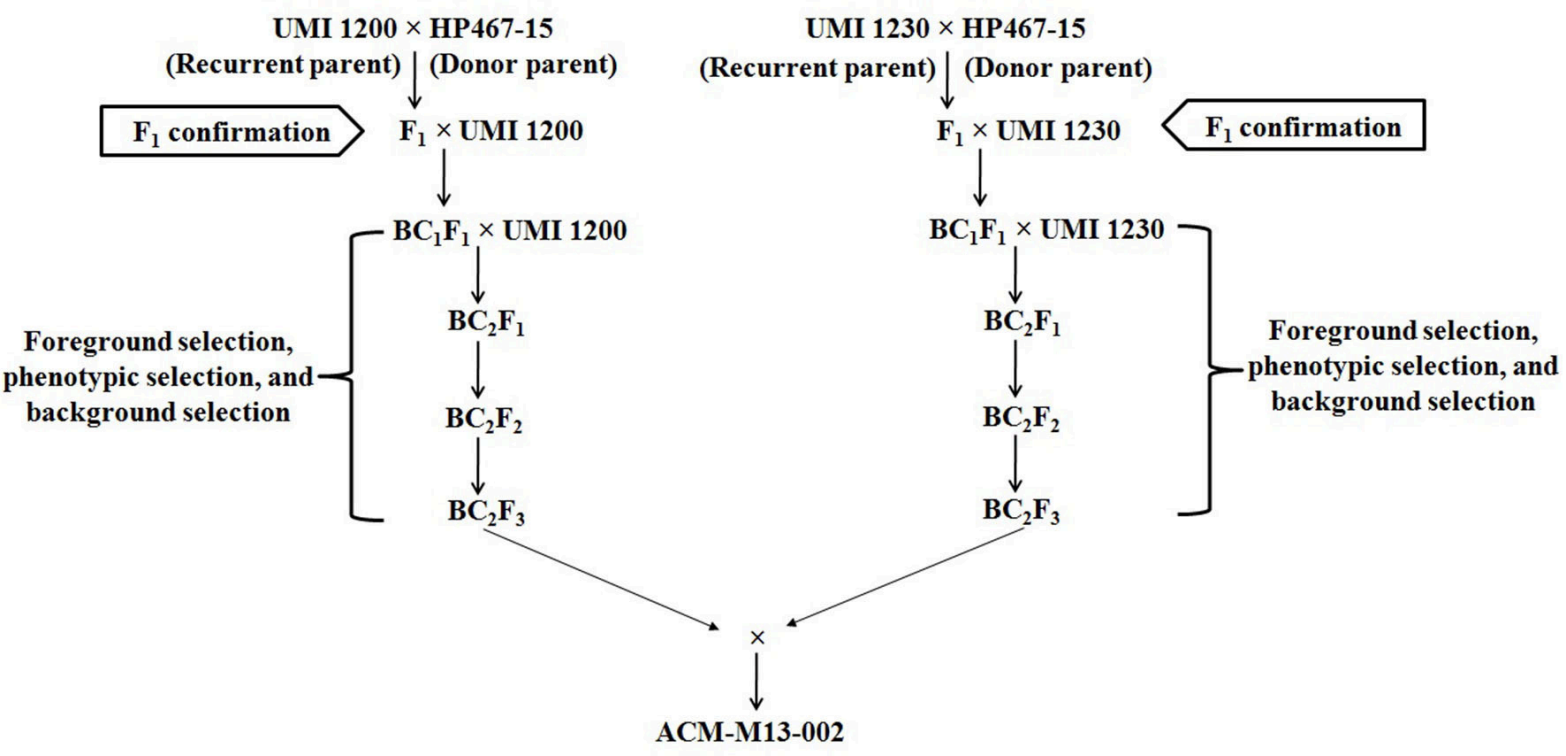
Scheme of marker assisted backcross breeding (MABB) for improving β-carotene concentration in parental lines of CO6 maize hybrid.

**Figure 2:**
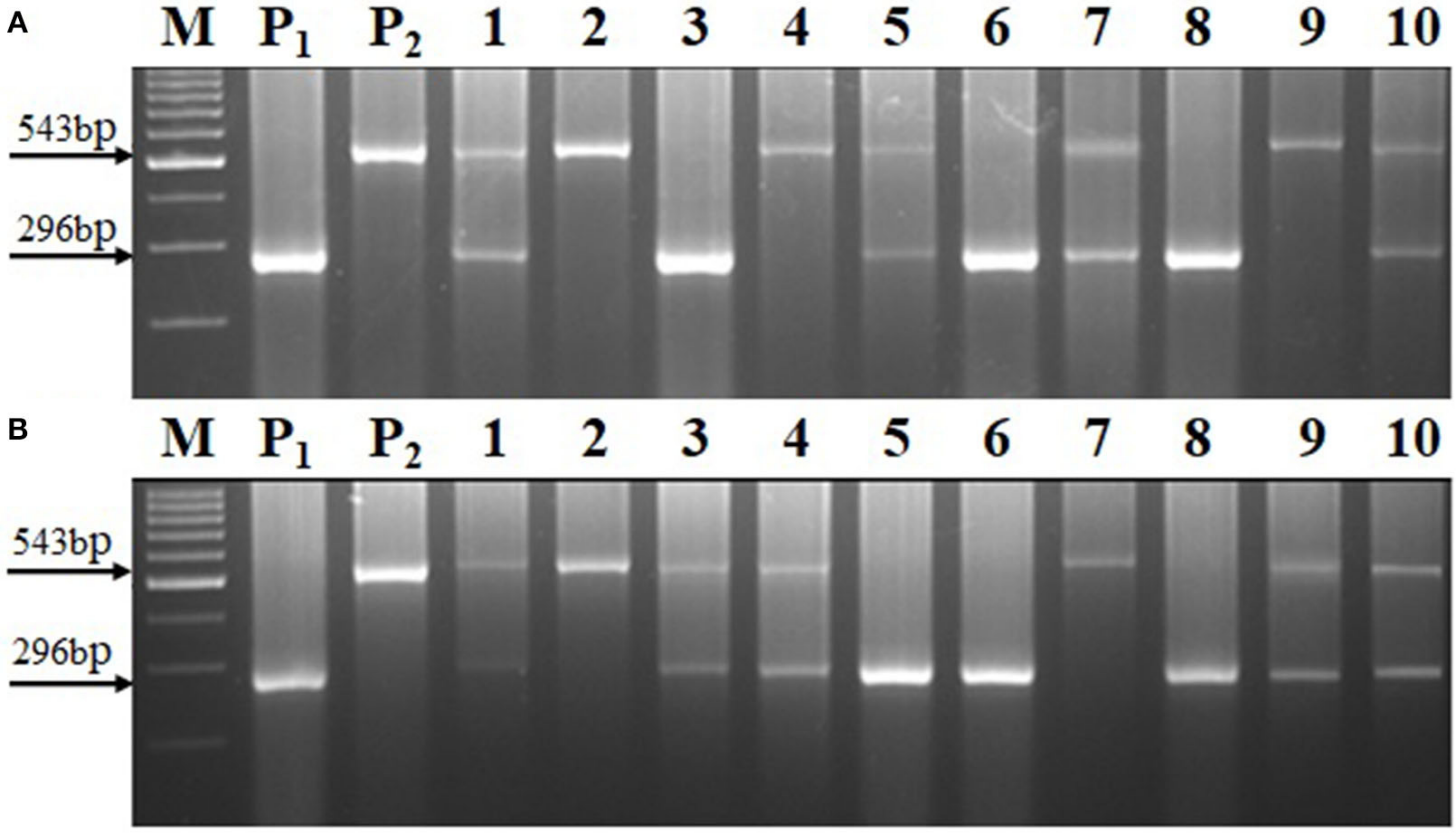
Segregation of allele1 and allele 3 in BC_2_F_2_ generation using the *crtRB1* gene specific marker (i.e., *crtRB1* 3′TE). **(A)** UMI1200 × HP467-15, **(B)** UMI1230 × HP467-15, **(M)** Ladder (100 bp), **(P**_**1**_**)** UMI1200/UMI1230, **(P**_**2**_**)** HP467-15, and **(1–10)** BC_2_F_2_ progenies.

**Figure 3:**
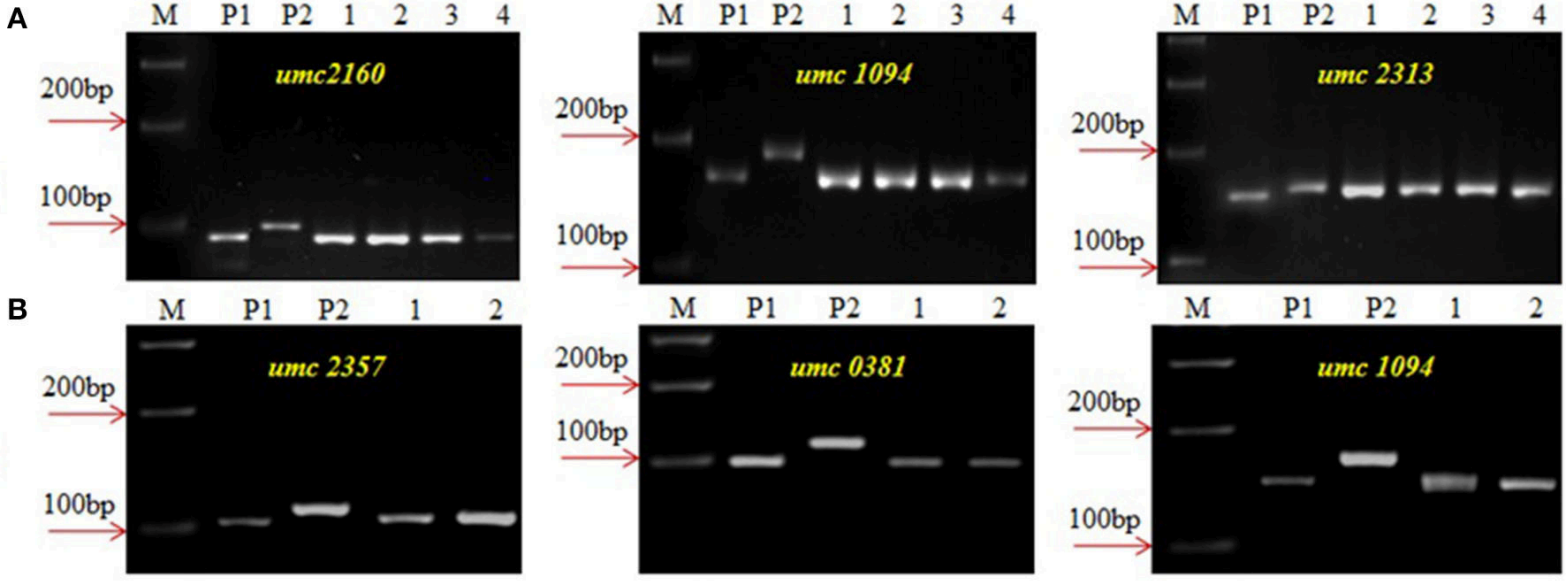
Background screening of improved lines using SSR markers. **(A)** UMI1200 × HP467-15, **(B)** UMI1230 × HP467-15, **(M)** Marker 100 bp, **(P**_**1**_**)** UMII1200/UMI1230, **(P**_**2**_**)** HP467-15, **(1–4)** Improved lines from UMI11200 × HP467-15 (1-UMI1200β^+^-1, 2-UMI1200β^+^-2, 3-UMI1200β^+^-3, 4-UMI1200β^+^-4, and **(1 and 2)** Improved lines from UMI1230 × HP467-15, (1-UMI1230β^+^-1, 2-UMI1230β^+^-2).

The *crtRB1* allele was introgressed into UMI1200 and UMI1230 using a recurrent backcrossing procedure, combined with foreground and background selection (Figure 1). Parental polymorphism screening was conducted between the recurrent and donor parents using a set of 214 SSR markers. In the cross combinations UMI1200 × HP467-15 and UMI1230 × HP467-15, among the 214 SSR markers, 117 and 109 SSR markers were found to be polymorphic markers. These polymorphic SSR markers were used for background selection and RPGR analysis. The F_1_ plants were produced from crosses UMI1200 × HP467-15 and UMI1230 × HP467-15. After confirming heterozygosity with a *crtRB1* 3'TE marker, the true F_1_ plants were backcrossed to their respective recurrent parent. The resulting BC_1_F_1_ population were screened with a *crtRB1* 3'TE marker and 24 heterozygous plants (allele 1/allele 3) in UMI1200 × HP467-15 and 37 heterozygous plants (allele 1/allele 3) in UMI1230 × HP467-15 were identified. The percentage of RPGR of positive plants ranged from 66.52–78.75% to 66.41–78.43%. The best BC_1_F_1_ plant showed a maximum RPGR of 78.75 and 78.43% was further selected to produce the BC_2_F_1_ population. In the BC_2_F_1_ population, a total of 53 and 87 plants were screened with a *crtRB1* 3'TE marker. Eleven heterozygous (allele 1/allele 3) plants in UMI1200 × HP467-15 and 18 heterozygous plants (allele 1/allele 3) in UMI1230 × HP467-15 were identified in the BC_2_F_1_ population. The percentage of RPGR of positive plants ranged from 81.39–88.12% to 82.59–89.65%. The best three BC_2_F_1_ plants from each cross having maximum RPGR were further selected and selfed to produce the BC_2_F_2_ population. A total of 146 and 126 BC_2_F_2_ plants were screened with a *crtRB1* 3'TE marker to identify homozygous plants (allele 1) (Figure 2). It revealed 34 homozygous plants in UMI1200 × HP467-15 and 31 homozygous plants in UMI1230 × HP467-15. The percentage of RPGR of positive plants ranged from 87.26–92.42% to 88.36–92.21%. The population details and their segregation pattern are presented in Table 1. The numbers of selected plants were further reduced to four and two based on the RPGR and phenotype data, and selfed to produce BC_2_F_3_ lines. As a result, four BC_2_F_3_ improved lines (UMI1200β^+^-1, UMI1200β^+^-2, UMI1200β^+^-3, and UMI1200β^+^-4) from the cross UMI1200 × HP467-15 and two BC_2_F_3_ improved lines (UMI1230β^+^-1 and UMI1230β^+^-2) from the cross UMI1230 × HP467-15 were developed. UMI1200 × HP467-15 based four BC_2_F_3_ lines showed 90.24–92.42% RPGR with an average of 91.33% and UMI1230 × HP467-15 based two BC_2_F_3_ lines showed 90.41% to 92.21% RPGR with an average of 91.31%. Among the improved lines, UMI1200β^+^-1 and UMI1230β^+^-1 showed the highest RPGR of 92.42% and 92.21% from their respective crosses (Figure 3). The postive plant's RPGR details are presented in Supplementary Tables 1, 2.

**Table 1:** Segregation pattern of alleles of the *crtRB1* gene in backcrossed and selfed progenies.

	**Cross**	**Generation**	**Population size**	**No of homozygotes (allele1/allele1)**	**No of heterozygotes (allele1/allele3)**	**No of homozygotes (allele3/allele3)**	***χ^2^***	***P*-value**
1	UMI1200 × HP467-15	BC_1_F_1_	100	–	24	76	27.04	0.0001^*^
		BC_2_F_1_	53	–	11	42	18.13	0.0001^*^
		BC_2_F_2_	146	34	46	66	33.99	0.0004^*^
2	UMI1230 × HP467-15	BC_1_F_1_	100	–	37	63	6.76	0.0093^*^
		BC_2_F_1_	87	–	18	69	29.89	0.0005^*^
		BC_2_F_2_	126	31	67	28	14.25	0.0008^*^

* *Significant at P < 0.05*.

### Introduction of *crtRB1* Gene Had No Effects on Agro-Morphological Characters

**Figure 4:**
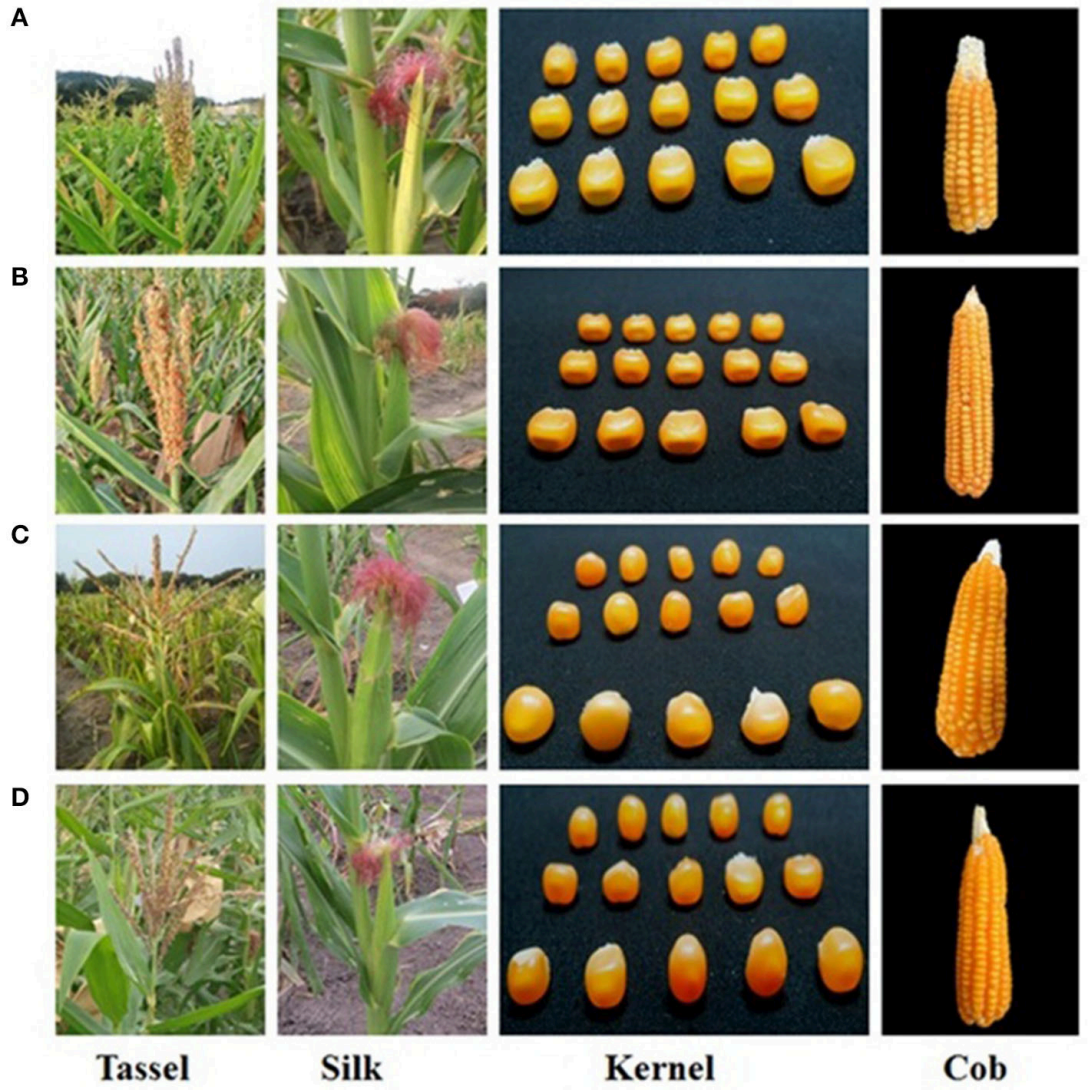
Morphological resemblance of the parents and the improved lines. **(A)** UMI1200, **(B)** UMI1200β^+^-1, **(C)** UMI1230, **(D)** UMI1230β^+^-1.

Six improved lines from both crosses were evaluated for the agro-morphological characters (Figure 4 and Table 2). It was revealed that all the improved lines recorded more than 80% of phenotypic resemblance, except for the number of kernel rows per cob (71.43%), to their respective recurrent parent. Among them, UMI1200β^+^-1, UMI1200β^+^-2, and UMI1200β^+^-4 from UMI1200 × HP467-15 and UMI1230 β^+^-1 from UMI1230 × HP467-15 recorded high phenotypic resemblance percentage (>90%) for more than six and five traits *viz.*, days to 50% tasselling, days to 50% silking, plant height, cob length, number of kernels per row, 100 kernel weight, and grain yield per plant. The overall performance showed that the traits recorded among the improved lines were on par with their recurrent parents and its phenotypic resemblance percentage ranging from 71.43% (number of kernel rows per cob) to 99.89% (grain yield per plant).

**Table 2:** β-carotene concentration and agronomic performance of the improved lines developed through MABB.

	**Genotypes**	**β-carotene (μg/g)**	**Plant height (cm)**	**Days to 50% tasseling (days)**	**Days to 50% silking (days)**	**Cob length (cm)**	**Number of kernel rows/cob**	**No of kernels/ row**	**100 kernel weight (g)**	**Mean grain yield/plant (g)**
1	^a^UMI1200β^+^-1	9.073 ± 0.557	126.50	58.00	61.00	13.10	12.00	10.00	24.10	87.12
2	^a^UMI1200β^+^-2	8.303 ± 0.364	126.10	59.00	59.00	13.40	10.00	10.00	24.10	87.32
3	^a^UMI1200β^+^-3	7.056 ± 0.562	126.80	57.00	61.00	13.20	12.00	09.00	24.20	87.61
4	^a^UMI1200β^+^-4	7.251 ± 0.453	126.30	58.00	60.00	13.50	12.00	10.00	24.60	88.12
5	^a^UMI1230β^+^-1	9.232 ± 0.508	133.90	57.00	59.00	12.10	10.00	09.00	23.89	76.41
6	^a^UMI1230β^+^-2	9.021 ± 0.471	134.10	56.00	58.00	12.30	10.00	08.00	23.78	76.39
7	^b^UMI1200 (RP)	0.893 ± 0.070	127.00	60.00	62.00	13.70	14.00	11.00	25.00	88.21
8	^b^UMI1230 (RP)	1.610 ± 0.083	135.00	58.00	60.00	12.50	12.00	10.00	24.10	76.71
9	^b^HP467-15 (DP)	10.525 ± 0.263	110.00	56.00	59.00	13.00	10.00	10.00	23.00	54.80
10	Test inbred mean	8.322 ± 0.485	128.95	57.50	59.67	12.93	11.00	9.33	24.11	83.83
11	Check inbred mean	4.342 ± 0.138	124.00	58.00	60.33	13.07	12.00	10.33	24.03	73.24

a Test inbreds and

b *Check inbreds*.

### β-Carotene Concentration in Improved Lines

The β-carotene concentration was estimated in six improved lines; it showed an increased level of β-carotene ranging from 7.056 to 9.232 μg/g with an average of 8.322 μg/g. The β-carotene concentration for the recurrent parents was 0.893 μg/g (UMI1200) and 1.610 μg/g (UMI1230). Introgression of allele1 resulted in an increased level of the kernel β-carotene concentration with a maximum of 9.073 μg/g in UMI1200β^+^-1 from UMI1200 × HP467-15 and 9.232μg/g in UMI1230β^+^-1 from UMI1230 × HP467-15. We also recorded a minimum level of the kernel β-carotene concentration 7.056 μg/g in UMI1200β^+^-3 and 9.021 μg/g in UMI1230β^+^-2. However, it was higher than their respective recurrent parent. The β-carotene concentration of improved lines is presented in Table 2. In addition, the β-carotene concentration of the improved lines at different environments are summarized in Supplementary Table 3.

### Evaluation of Hybrid Combination Under Comparative Yield Trial (CYT) and Multi-Location Trial (MLT)

**Figure 5:**
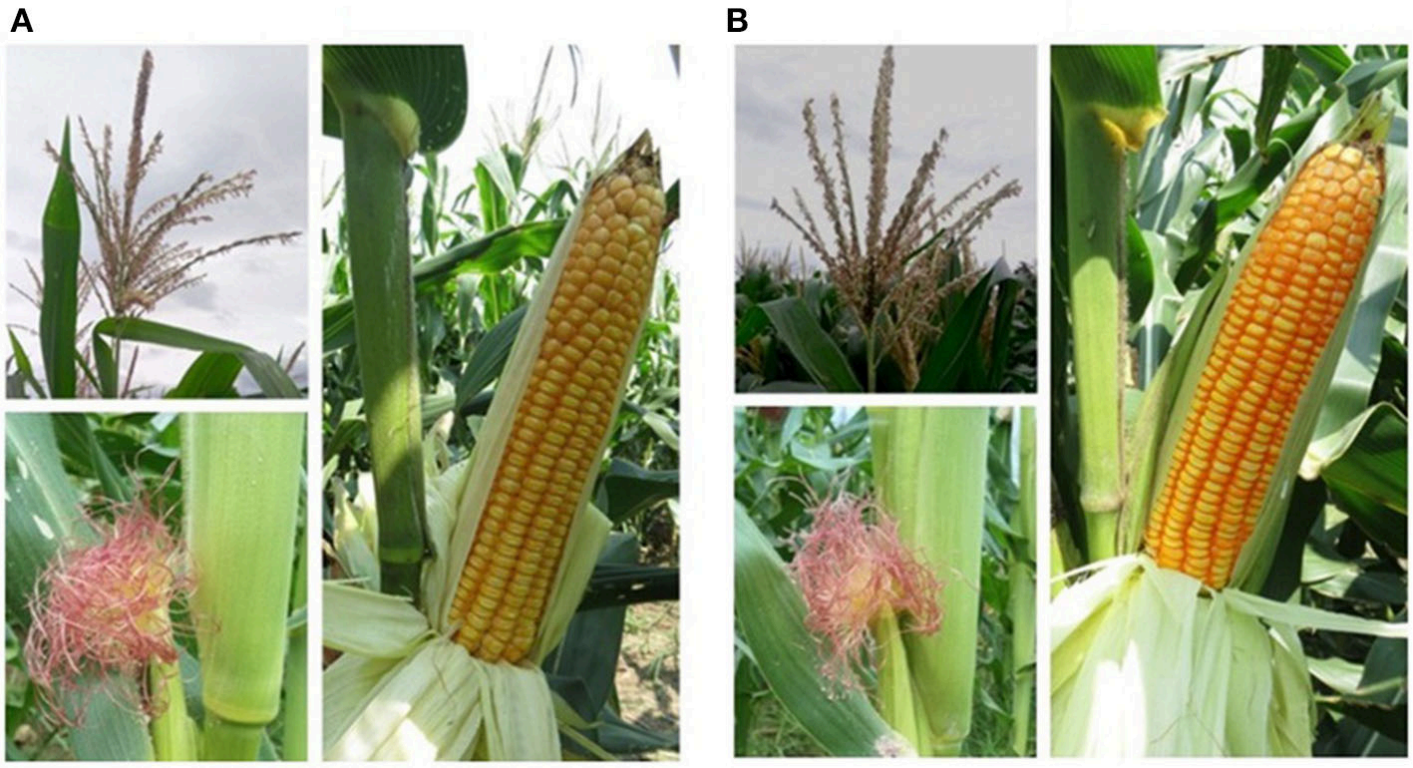
Morphological characteristics of original version and reconstituted hybrid with high β-carotene. **(A)** CO6, **(B)** ACM-M13-002.

Five hybrid combinations were developed using the improved lines. The agronomic performance and the β-carotene concentration of the five hybrids were evaluated along with their corresponding original hybrid (CO6) and a commercial hybrid (NK6240) under CYT (Figure 5). Among the five hybrids, ACM-M13-002 showed a 13.38% yield increase and a 7.3-fold β-carotene increase over its original hybrid and 15.59% yield increase over a commercial hybrid NK6240 (Table 3). Thus, ACM-M13-002 forwarded to MLT along with eight hybrids to study the GE interaction over the different maize growing regions in Tamil Nadu. The yield performance of MLT showed that the hybrid ACM-M13-002 (G3) recorded a value that is comparable to that of its original hybrid (CO6) in all the locations. An average yield performance across 10 environments recorded 7,256.2 kg/ha, which was comparable with the original hybrid CO6 (G2) (7,358.1 kg/ha).

**Table 3:** Agronomic performance and β-carotene value of hybrids developed through MABB under comparative yield trail (CYT).

**Hybrid**	**Plant height (cm)**			**Days to 50% tasseling**			**Days to 50% silking**			**1,000 grain weight (g)**			**Grain yield/plant (g)**					**Yield (t/ha)**	**β-carotene (μg/g)**
	**VGD^1^ Rabi 2014–15**	**VGD^1^ Kharif 2015**	**VGI^2^ Rabi 2014–15**	**VGD^1^ Rabi 2014–15**	**VGD^1^ Kharif 2015**	**VGI^2^ Rabi 2014–15**	**VGD^1^ Rabi 2014–15**	**VGD^1^ Kharif 2016**	**VGI^2^ Rabi 2014–15**	**VGD^1^ Rabi 2014–15**	**VGD^1^ Kharif 2015**	**VGI^2^ Rabi 2014–15**	**VGD^1^ Rabi 2014–15**	**VGD^1^ Kharif 2015**	**VGI^2^ Rabi 2014–15**	**% increase over CO6**	**% increase over NK6240**		
^a^ACM-M13-001^3^	185.00	184.00	186.00	46.00	47.00	46.00	48.00	49.00	49.00	345.00	343.00	344.00	163.00	164.00	164.00	4.49	6.53	10.93	8.92
^a^ACM-M13-002^3^	187.00	186.00	188.00	45.00	46.00	45.00	48.00	48.00	47.00	330.00	329.00	332.00	179.00	176.00	178.00	13.38	15.59	11.86	9.05
^a^ACM-M13-003^3^	238.00	237.00	239.00	50.00	49.00	49.00	53.00	52.00	52.00	400.00	401.00	400.00	160.00	162.00	162.00	2.58	4.58	10.73	8.24
^a^ACM-M13-004^3^	206.00	205.00	206.00	48.00	49.00	48.00	51.00	52.00	51.00	420.00	420.00	418.00	164.00	162.00	163.00	3.82	5.84	10.86	8.47
^a^ACM-M13-005^3^	208.00	207.00	208.00	49.00	49.00	48.00	52.00	52.00	50.00	340.00	339.00	341.00	161.00	162.00	160.00	2.58	4.58	10.73	8.83
SE^4^	9.55	9.55	9.53	0.93	0.63	0.73	1.03	0.87	0.86	18.00	18.39	17.49	3.47	2.73	3.22	2.04	2.08	0.21	0.15
SD^5^	21.35	21.35	21.30	2.07	1.41	1.64	2.30	1.95	1.92	40.25	41.13	39.12	7.77	6.10	7.20	4.55	4.64	0.48	0.33
^b^CO6^6^	189.00	188.00	190.00	53.00	53.00	52.00	56.00	56.00	55.00	400.00	401.00	400.00	157.00	159.00	157.00			10.46	1.24
^b^NK6240^6^	200.00	198.00	200.00	49.00	50.00	48.00	52.00	53.00	51.00	330.00	331.00	330.00	168.00	169.00	167.00			10.26	0.71
Test hybrid mean	204.80	203.80	205.40	47.60	48.00	47.20	50.40	50.60	49.80	367.00	366.40	367.00	165.40	165.20	165.40	5.37	7.42	11.02	8.7
Check hybrid mean	194.50	193.00	195.00	51.00	51.50	50.00	54.00	54.50	53.00	365.00	366.00	365.00	162.50	164.00	162.00			10.36	0.97

1 *Vaigai dam*,

2 *Vagarai*,

3 *High beta carotene hybrids*,

4 *Standard error*,

5 *Standard deviation*,

6 *Standard checks*.

a *Test hybrids*,

b *Check hybrids. Note: Yield (t/ha) given is the average of the hybrids*.

**Figure 6:**
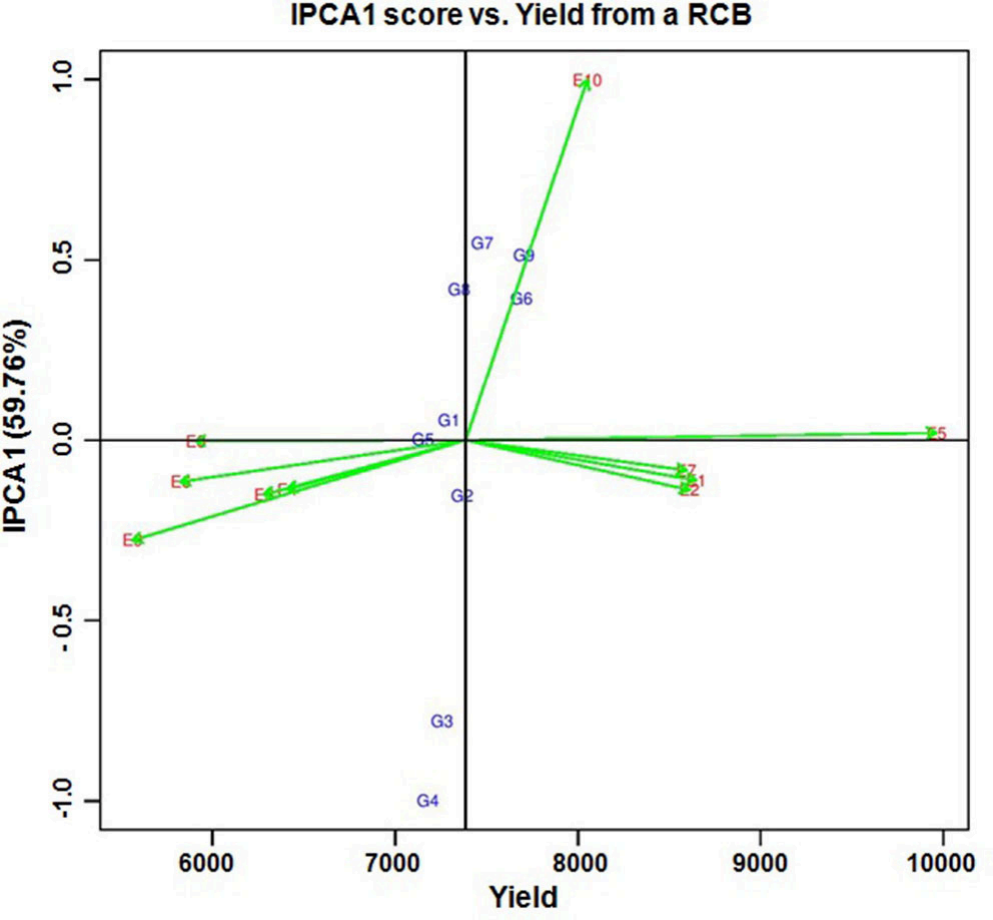
AMMI biplot for IPCA1 score vs. yield in nine hybrids of maize from 10 environments in the state of Tamil Nadu. ***(G1)*** *NK6240*, ***(G2)*** *CO 6*, ***(G3)*** *ACM-M13-002*, ***(G4)*** *ACM-M13-007*, ***(G5)*** *CMH 11-583*, ***(G6)*** *VaMH 14020*, ***(G7)*** *VaMH 12014*, ***(G8)*** *900 M GOLD, and* ***(G9)*** *CMH 11-586*. ***(E1)*** *Coimbatore*, ***(E2)*** *Vagarai*, ***(E3)*** *Vridhachalam*, ***(E4)*** *Bhavanisagar*, ***(E5)*** *Coimbatore*, ***(E6)*** *Vagarai*, ***(E7)*** *Vridhachalam*, ***(E8)*** *Bhavanisagar*, ***(E9)*** *Vaigai dam, and* ***(E10)*** *Athiyandal*.

**Figure 7:**
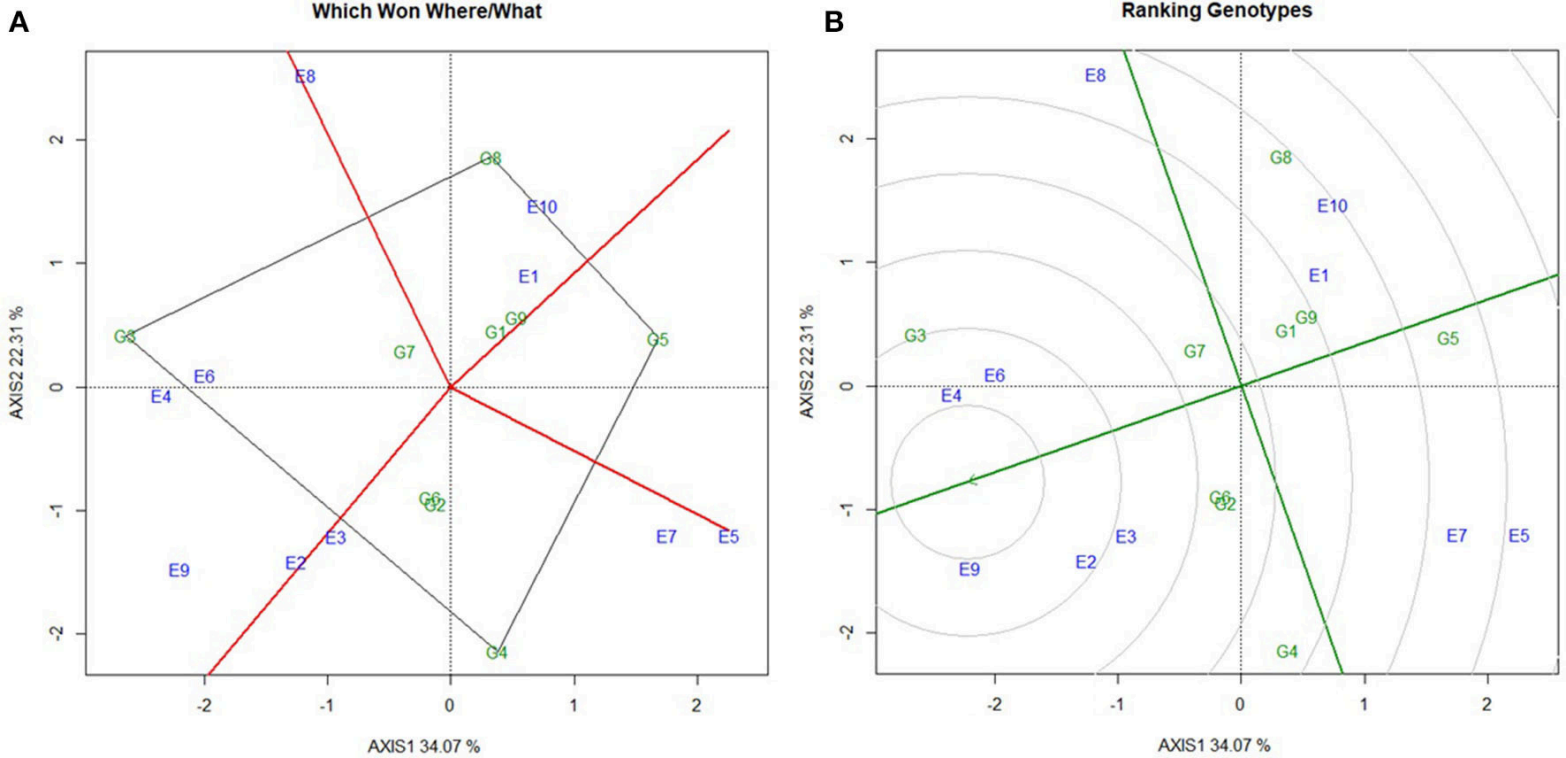
Stability analysis to evaluate the mean grain yield of nine maize hybrids. **(A)** Polygon view of GGE biplot for grouping the environments, **(B)** GGE biplot for comparing the test hybrids with ideal environment. ***(G1)*** *NK6240*, ***(G2)*** *CO 6*, ***(G3)*** *ACM-M13-002*, ***(G4)*** *ACM-M13-007*, ***(G5)*** *CMH 11-583*, ***(G6)*** *VaMH 14020*, ***(G7)*** *VaMH 12014*, ***(G8)*** *900 M GOLD*, ***(G9)*** *CMH 11-586*, ***(E1)*** *Coimbatore*, ***(E2)*** *Vagarai*, ***(E3)*** *Vridhachalam*, ***(E4)*** *Bhavanisagar*, ***(E5)*** *Coimbatore*, ***(E6)*** *Vagarai*, ***(E7)*** *Vridhachalam*, ***(E8)*** *Bhavanisagar*, ***(E9)*** *Vaigai dam, and* ***(E10)*** *Athiyandal*.

*Per se* performance of the yield for the developed five hybrids ranged from 10.73 to 11.86 (t/ha) with an average of 11.02 (t/ha). Analysis of variation for the grain yield (kg/ha) of maize hybrids showed a significant difference between the components under study (Genotypes, Environments and Genotype × Environment) with the percent accounting for the highest total variation sum of the square by 80% for environments, 1.33%, and 18.68% for GE (Tables 4, 5 and Supplementary Table 4). The effect of the genotype and environment is 14.04 times higher compared to the component genotype. Hence, the variation that exists among the genotypes across the environments paves the way to understanding the yield stability of the hybrids over the varying environments and specific adaption of the hybrids. Based on the Interaction Principal Component Axis 1 (IPCA1) of the AMMI model, the genotypes G1, G2, and G5 recorded values nearer to the zero on the biplot (0.056, −0.154, and 0.002) which represents that the genotypes are highly stable with good yield potential (Figure 6). From the “Which won where” polygon view of GGE analysis (Figure 7) four genotypes - G3, G4, G5, and G8 - were located at the corner, which denotes that the genotypes are specifically adapted to the particular environment for the trait under study. Average Environment Coordination (AEC) recorded that G3 is placed at the second concentric circle and is ideal in terms of high yielding ability and stability, whereas other genotypes, G2, G6, and G7, placed at the third and fourth concentric circle where the stability gradient is low compared to the genotypes closer to the center axis. Based on the vertices of the Average Environment Axix (AEA), the environments E9 and E4 were considered as highly interactive environments from where the wider adaption of the genotypes are selected.

**Table 4:** Analysis of variance of yield data of nine maize hybrids tested across environments during Kharif 2016 and 2017.

**S.No**	**Source**	**DF**	**SS**	**MS**	**Total variation explained (%)**
1	Environment (E)	9	391935207	43548356^**^	80.00
2	Genotype (G)	8	6505899	813237^**^	1.33
3	Genotype × Environment Interaction (GE)	72	91495843	1270776^**^	18.68
4	PC1	16	54822906	3426432^**^	59.76
5	PC2	14	15431914	1102280^**^	16.82
6	PC3	12	8193433	682786^**^	8.93
7	Residuals	90	2241107	24901^**^	0.00

** *Represents highly significant at 1% level*.

**Table 5:** Multi location trial (MLT) conducted in Kharif 2016 and 2017.

**S. No**	**Hybrid**	**Parents**	**Code for hybrids**	**Grain yield (Kg/ha) kharif 2016**				**Grain yield (Kg/ha) kharif 2017**						
				**CBE^**E1**^**	**VGI^**E2**^**	**VRI^**E3**^**	**BSR^**E4**^**	**CBE^**E5**^**	**VGI^**E6**^**	**VRI^**E7**^**	**BSR^**E8**^**	**VGD^**E9**^**	**CEM^**E10**^**	**Mean**
1	NK6240	–	G1	8,705.0	8,598.5^*^	5,508.0	6,564.5	10,447.5	5,023.5	8,220.5	6,141.5	5,671.5	8,347.0	7,322.8
2	CO6	UMI1200 × UMI1230	G2	8,796.5	9,466.5	5,912.0	6,008.5	10,125.0	6,12.0	8,381.5	5,554.0	5,794.0^*^	7,531.0	7,358.1
3	ACM-M13-002	UMI1200β^+^ × UMI1230β^+^	G3	8,794.5	8,824.0	6,875.5^*^	7,138.5^*^	8,309.0	6,452.5	7,615.5	6,393.5^*^	6,393.5^*^	5,765.0	7,256.2
4	ACM-M13-007	UMI285lpa × UMI1200-1	G4	8,477.5	8,474.5	6,681.5^*^	6,493.5	10,525.0	5,715.0	9,499.0^*^	5,225.5	6,005.5^*^	4,693.5	7,179.1
5	CMH 11-583	N09-153-3A × N10-65-3	G5	8,984.5^*^	8,264.0	5,918.0	5,563.0	10,349.5	5,478.5	8,892.0^*^	5,745.0	4,730.0	7,622.0	7,154.7
6	VaMH 14020	UMI1230 × VIM153	G6	8,147.0	8,850.0	6,882.5^*^	6,557.0	10,258.0	6,002.0^*^	9,008.0^*^	5,702.0	5,837.5^*^	9,664.0^*^	7,690.8
7	VaMH12014	UMI1200 × VIM357	G7	7,507.0	8,574.5	6,324.0	6,695.0^*^	9,766.5	6,070.5^*^	8,583.5	6,041.5	5,305.0	9,876.0^*^	7,474.4
8	900 M GOLD	-	G8	8,989.0^*^	8,127.5	5,110.0	6,255.0	9,249.5	6,118.0^*^	8,913.5^*^	6,393.5^*^	5,094.0	9,263.5^*^	7,351.4
9	CMH 11-586	N09-164-2 × N148	G9	9,435.5	8,319.5	7,333.5^*^	6,416.5	10,618.0^*^	5,550.5	8,235.5	5,978.5	5,237.5	9,924.0^*^	7,704.9
10	^a^SEd			140.37	115.25	87.36	116.95	257.63	65.31	110.11	133.76	82.26	138.49	
11	^b^CD (*p* = 0.05)			323.71	265.76	201.46	269.68	594.11	150.60	253.92	308.45	189.70	319.37	
12	Mean			8,648.5	8,611.0	6,282.8	6,410.8	9,960.9	5,824.7	8,594.3	5,908.3	5,563.8	8,076.2	

***(E1)** Coimbatore, **(E2)** Vagarai, **(E3)** Vridhachalam, **(E4)** Bhavanisagar, **(E5)** Coimbatore, **(E6)** Vagarai, **(E7)** Vridhachalam, **(E8)** Bhavanisagar, **(E9)** Vaigai dam, and **(E10)** Athiyandal*,

a *Standard error deviation*,

b *Critical difference*.

* *Represents significant at 5% level*.

## Discussion

CO6 is one of the popular maize hybrids of India, especially in Tamil Nadu. CO6 is known for its high yield, multiple disease resistances, and its suitablity for rainfed and irrigated conditions. However, CO6 is low in pVAC β-carotene, thus, the present investigation was taken up to improve the β-carotene in CO6 through MABB. Previously, Muthusamy et al. (9) and Zunjare et al. (10) used an accelerated MABB scheme to generate the improved version of VQL1-K10-40-11-53 × VQL2-K10-08-14, HQPM7-B, HQPM 5-C hybrids for β-carotene. We also used the same MABB scheme to enrich the β-carotene in CO6. Marker-assisted foreground selection using gene-linked markers permits the transfer of the gene of interest with high precision in MABB. The *crtRB1* gene-specific marker (*crtRB1* 3′TE) used in this study allows us to detect the positive plants exactly without any false positives at any stage of MABB. The advantages of using a linked marker for the selection of single or multiple genes simultaneously in MABB have been described in many studies for nutritional traits in maize (7, 8, 13). The backcross and selfed-generations from both crosses, UMI1200 × HP467-15 and UMI1230 × HP467-15, showed segregation distortion. The segregation pattern of allele 1 and allele 3 deviated from the expected Mendelian ratio (1:1 and 1:2:1) and allele 1 was diminished. These results are similar to the reports of Babu et al. (8), who described the segregation distortion of allele 1 in five out of eight populations. There are many facts, such as embryo-specific mutation (24), segregation distortion regions in the maize genome (25), mutants like the defective kernel (24), gametophytic factors (24, 26), and genetic background of the target allele (8), that are reported to be the reason for the segregation distortion. In this case, the evaluation of a large population for attaining enough foreground positives is essential. Marker-assisted background selection using SSR markers helped in selecting the foreground positive plants with a high RPGR. We performed the background selection starting from the BC_1_F_1_ generation; it hastened the RPGR in advanced lines. Following this approach, after only two generations of backcrossing, it was possible to identify the plants carrying RPGR ranging from 90.24 to 92.42% (UMI1200 × HP467-15) and 90.41–92.21% (UMI 1230 × HP467-15) in BC_2_F_3_ improved lines. These results are in accordance with the previous studies (9, 27, 28).

The study aims to introgress the *crtRB1* gene without troubling the recurrent parental genome by MABB, thus the improved line will be suitable for further use. For confirming the suitability of the new genotypes, it is essential to characterize and choose the progenies that are closer to the parent for both morphological and nutritional traits as this selection adds value to the MABB program. Thus, we investigated the agro-morphological characters of the improved lines. It revealed that the agro-morphological characters of the improved lines were on par with the recipient parent. The agro morphological characters among the six improved lines ranged from 71.43 to 99.89%. Among them, UMI1200β^+^-1, UMI1200β^+^-2, and UMI1200β^+^-4 from UMI1200 × HP467-15, and UMI1230β^+^-1 from UMI1230 × HP467-15 recorded more than 90% recovery for the traits *viz.*, days to tasseling, days to silking, plant height, cob length, number of kernels per row, 100 kernel weight, and grain yield per plant. Moreover, the β-carotene content of the improved lines ranged from 7.056 to 9.073 μg/g (UMI1200 × HP467-15) and 9.021–9.232 μg/g (UMI1230 × HP467-15). The β-carotene concentration of the improved lines is on par with the β-carotene parent. Similar results were obtained in other studies (10, 29).

The improved lines can be considered as the candidate parents for developing β-carotene hybrids adapted to irrigated and rainfed conditions. They were successfully used to develop the five hybrid combinations. Among them, ACM-M13-002 is a superior hybrid with increased yield and β-carotene concentration over its original hybrid and a commercial hybrid NK6240 under CYT. Hence, this hybrid was forwarded for MLT along with eight hybrids to study the GE interaction over the different maize growing regions in Tamil Nadu. The yield performance of MLT showed that ACM-M13-002 (G3) recorded a value that is comparable to that of its original hybrid CO6 (G2) in all the locations. Based on the stability ANOVA, the GE toward the yield was lesser compared to the environment (E) and it is 14.04 times higher than that of genotype (G). These results are similar to the earlier study on maize (20). We observed significant genotype by environment interaction (GE) effect, which revealed that the yield performance of all the hybrids over the environments under study was not consistent. This situation necessitates understanding the nature and magnitude of genotype by environment effect which may not be possible through standard ANOVA (21, 22). IPCA1 from the AMMI model gives an idea about the association of the genotypes with the environments under study. A higher IPCA1 value reflects the specific adaptation of the genotypes irrespective of the direction in which it falls (Positive/ Negative) and the values nearer to the zero reflect the genotypes widely adapted to all the environments tested. From the above concept the genotype G1, G2, and G5 recorded values closer to the zero, which could be promising genotypes for the yield trait over all the environments. GGE biplot analysis reflects the similarity existing between the genotypes, environment, and their corresponding response to each other, as well as the interaction between them, making it the best tool to graphically represent the stability of the genotype (23). From the polygon view of GGE biplot, the genotypes G3, G4, G5, and G8 are recorded as a specific adaptation to the respective environments which are present nearer to the vertex of the polygon. High *per se* performance, higher vector length, and high stability across the environments are the properties of ideal genotype and the genotypes places closed to the ideal genotype are called desirable genotypes. These are most probably at the second concentric circle of the GGE biplot (23), with the greatest ability to discriminate genotypes, favoring the selection of superior genotypes. The GGE biplot revealed that the genotype G3 (ACM-M13-002) could be recommended for yield trait to all the environments studied since it is placed nearer to the highly interactive environment (E4) and also at the second concentric circle on the GGE biplot. In summary, we have successfully improved the β-carotene concentration in the CO6 hybrid through MABB. The β-carotene enriched hybrid developed in this study will hold great promise for nutritional and food security. Also, the improved maize inbreds with β-carotene will serve as a potential genetic material for the development of β-carotene rich cultivars in maize breeding programs.

## Data Availability Statement

The datasets generated for this study are available on request to the corresponding author.

## Author Contributions

SN conceived and designed the methods and experiments. SN, TD, GK, NT, and RR constructed backcross progenies and managed field work. TD, BP, SC, JN, DM, KA, and VS conducted phenotype, genotype, and biochemical analyses. SN, BP, SC, and DM analyzed the data. LM and SM provided suggestions on experiments and data analysis. SN, KA, DM, and SC drafted the manuscript. All authors contributed to the article and approved the submitted version.

## Conflict of Interest

The authors declare that the research was conducted in the absence of any commercial or financial relationships that could be construed as a potential conflict of interest.
