# Supplementary material for: Enhancing β-Carotene Concentration in Parental Lines of CO6 Maize Hybrid Through Marker-Assisted Backcross Breeding (MABB)
Source: Front Nutr. 2020 Oct 14;7:134. doi: 10.3389/fnut.2020.00134 (PMC7591750; doi:10.3389/fnut.2020.00134)
Supplement: Supplementary file 1 [file Data_Sheet_1.doc]

**Table S1 |** Details of background analysis in UMI1200 × HP467-15.

| **S.No** | **Generation** | **Number of plants** | | | **Back ground analysis** | | |
| --- | --- | --- | --- | --- | --- | --- | --- |
|  |  | **Total** | **Fore ground positives** | **^a^Selected** | **^b^Total markers** | **Markers recovered** | **Recovery percentage**  **(%)** |
| 1 | BC_1_F_1_ | 100 | 24 | 1 | 117 | 65 - 77 | 66.52– 78.75 |
| 2 | BC_2_F_1_ | 53 | 11 | 3 | 117 | 83 - 89 | 81.39 – 88.12 |
| 3 | BC_2_F_2_ | 146 | 34 | 4 | 117 | 88 - 101 | 87.26 –92.42 |
| 4 | BC_2_F_3_ | 4 | 4 | 4 | 117 | 96 - 101 | 90.24 – 92.42 |

*Note: ^a^Total number of plants forwarded to successive generation, ^b^Total number of polymorphic markers.*

**Table S2 |** Details of background analysis in UMI1230 × HP467-15.

| **S.No** | **Generation** | **Number of plants** | | | **Back ground analysis** | | |
| --- | --- | --- | --- | --- | --- | --- | --- |
|  |  | **Total** | **Fore ground positives** | **^a^Selected** | **^b^Total markers** | **Markers recovered** | **Recovery percentage**  **(%)** |
| 1 | BC_1_F_1_ | 100 | 37 | 1 | 109 | 57 - 72 | 66.41 – 78.43 |
| 2 | BC_2_F_1_ | 87 | 18 | 3 | 109 | 78 - 88 | 82.59– 89.65 |
| 3 | BC_2_F_2_ | 126 | 31 | 2 | 109 | 87 - 93 | 88.36 – 92.21 |
| 4 | BC_2_F_3_ | 2 | 2 | 2 | 109 | 91 - 93 | 90.41 – 92.21 |

*Note: ^a^Total number of plants forwarded to successive generation, ^b^Total number of polymorphic markers.*

**Table S3 |** β-carotene concentration of the improved lines at five different environments

| **S.No** | **Parental lines** | **VGD^1^**  **Rabi 2014-2015** | **VGI^2^**  **Rabi 2014-2015** | **VGD^1^**  **Kharif 2015** | **VGD^1^**  **Rabi 2015-16** | **VGD^1^**  **Kharif 2016** |
| --- | --- | --- | --- | --- | --- | --- |
| 1 | ^a^UMI1200β^+^-1 | 9.071 | 8.827 | 9.077 | 8.992 | 9.037 |
| 2 | ^a^UMI1200β^+^-2 | 8.371 | 8.134 | 8.298 | 8.306 | 8.388 |
| 3 | ^a^UMI1200β^+^-3 | 7.041 | 7.073 | 7.013 | 7.134 | 7.023 |
| 4 | ^a^UMI1200β^+^-4 | 7.261 | 7.199 | 7.259 | 7.267 | 7.245 |
| 5 | ^a^UMI1230β^+^-1 | 9.245 | 9.276 | 9.230 | 9.225 | 9.241 |
| 6 | ^a^UMI1230β^+^-2 | 9.045 | 9.015 | 9.037 | 9.156 | 9.114 |
| 7 | SE^3^ | 0.39 | 0.38 | 0.39 | 0.38 | 0.40 |
| 8 | SD^4^ | 0.96 | 0.94 | 0.97 | 0.94 | 0.98 |
| 9 | Test inbred mean | 8.339 | 8.254 | 8.319 | 8.346 | 8.341 |
| 10 | ^b^UMI1200 | 0.891 | 0.754 | 0.658 | 0.789 | 0.854 |
| 11 | ^b^UMI1230 | 1.356 | 1.558 | 1.611 | 1.499 | 1.654 |
| 12 | Check inbred mean | 1.123 | 1.156 | 1.134 | 1.144 | 1.254 |

*^1^Vaigai Dam, ^2^Vagarai, ^3^Standard error, ^4^Standard deviation, ^a^Test inbreds, ^b^Check inbreds.*

*Note: All the values presented has represented by the unit of μg/g concentration.*

**Table S4 |** ANOVA for yield traits of nine hybrids at ten different environments

| **Source  of variation** | **DF** | **Mean Sum of Square** | | | | | | | | | |
| --- | --- | --- | --- | --- | --- | --- | --- | --- | --- | --- | --- |
|  |  | **E1** | **E2** | **E3** | **E4** | **E5** | **E6** | **E7** | **E8** | **E9** | **E10** |
| Total | 17 | 300935.53 | 159352.47 | 500310.59 | 191100.23 | 563674.38 | 177423.06 | 294211.75 | 151872.00 | 253782.59 | 3303988.75 |
| Replication | 1 | 1464.89 | 15473.78 | 448.00 | 5084.44 | 71.11 | 69816.89 | 412.44 | 20963.55 | 35463.11 | 123036.45 |
| Treatment | 8 | 619600.00** | 323408.00** | 1055472.00** | 391776.00** | 1131424.00** | 364032.00** | 613024.00** | 302216.00** | 528088.00** | 6986416.00** |
| Error | 8 | 19704.89 | 13281.78 | 7632.00 | 13676.44 | 66375.11 | 4264.89 | 12124.44 | 17891.55 | 6767.11 | 19180.45 |

*Note: **Significance at the level of 1%,* ***(DF)*** *Degrees of freedom,* ***(E1)*** *Coimbatore,* ***(E2)*** *Vagarai,* ***(E3)*** *Vridhachalam ,* ***(E4)*** *Bhavanisagar,* ***(E5)*** *Coimbatore,* ***(E6)*** *Vagarai,* ***(E7)*** *Vridhachalam,* ***(E8)*** *Bhavanisagar,* ***(E9)*** *Vaigai Dam and* ***(E10)*** *Athiyandal.*

\
